# Dual Regulation Mechanism of Obesity: DNA Methylation and Intestinal Flora

**DOI:** 10.3390/biomedicines12081633

**Published:** 2024-07-23

**Authors:** Yi Ren, Peng Huang, Lu Zhang, Yu-Fen Tang, Sen-Lin Luo, Zhou She, Hong Peng, Yu-Qiong Chen, Jin-Wen Luo, Wang-Xin Duan, Ling-Juan Liu, Li-Qun Liu

**Affiliations:** 1 Department of Pediatrics, The Second Xiangya Hospital of Central South University, Changsha 410011, China; renwing1981@163.com (Y.R.); huangpeng202202@163.com (P.H.); zl8212224@163.com (L.Z.); tangyu9880@163.com (Y.-F.T.); senlinluo@csu.edu.cn (S.-L.L.); shezhou1996@163.com (Z.S.); ph218212222@163.com (H.P.); yuqiongrabbit@163.com (Y.-Q.C.); luojinwen990311@163.com (J.-W.L.); wd10010707@outlook.com (W.-X.D.); liulingjuan@csu.edu.cn (L.-J.L.); 2 Children’s Brain Development and Brain Injury Research Office, The Second Xiangya Hospital of Central South University, Changsha 410011, China; 3 Department of Pediatrics, Haikou Hospital of the Maternal and Child Health, Haikou 570100, China; 4 Department of Children’s Healthcare, Hainan Modern Women and Children’s Medical, Haikou 570100, China

**Keywords:** obesity, intestinal flora, DNA methylation, epigenetics

## Abstract

Obesity is a multifactorial chronic inflammatory metabolic disorder, with pathogenesis influenced by genetic and non-genetic factors such as environment and diet. Intestinal microbes and their metabolites play significant roles in the occurrence and development of obesity by regulating energy metabolism, inducing chronic inflammation, and impacting intestinal hormone secretion. Epigenetics, which involves the regulation of host gene expression without changing the nucleotide sequence, provides an exact direction for us to understand how the environment, lifestyle factors, and other risk factors contribute to obesity. DNA methylation, as the most common epigenetic modification, is involved in the pathogenesis of various metabolic diseases. The epigenetic modification of the host is induced or regulated by the intestinal microbiota and their metabolites, linking the dynamic interaction between the microbiota and the host genome. In this review, we examined recent advancements in research, focusing on the involvement of intestinal microbiota and DNA methylation in the etiology and progression of obesity, as well as potential interactions between the two factors, providing novel perspectives and avenues for further elucidating the pathogenesis, prevention, and treatment of obesity.

## 1. Introduction

**Figure 1:**
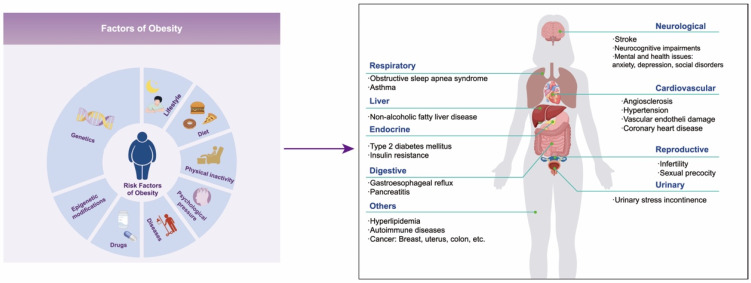
The left diagram shows the factors of obesity, and the right shows the complications of obesity.

Obesity is a chronic metabolic disorder caused by excessive accumulation or abnormal distribution of fat in the body, and its global prevalence rate is increasing year by year. In 2016 alone, over 650 million people worldwide suffered from obesity [1]. The World Obesity Federation estimates that in 2030, 254 million children and adolescents aged 5–19 will be obese, and approximately 58% of adults globally will be overweight or obese [2]. Obesity is the driving factor of numerous diseases that not only seriously affect the quality of life of patients but also shorten their life expectancy. These diseases include metabolic disorders (such as type 2 diabetes mellitus and hyperlipidemia), non-alcoholic fatty liver disease, cardiovascular diseases, hypertension, chronic kidney disease, autoimmune diseases, neurocognitive impairments, mental and health issues (such as anxiety, depression, and social disorders), cancers, etc. (Figure 1) [3,4,5,6]. It is reported that individuals aged 20–29 with obesity or severe obesity may experience a reduction in life expectancy of 5.6–10.3 years [7]. Therefore, the health risks associated with obesity have emerged as a significant public health problem that needs to be solved urgently around the world.

Obesity is a multi-factor disease influenced by genetic, environmental, and other factors [8]. The genetic susceptibility to obesity accounts for up to 75% [9,10], which is the major reason for the individual differences in the development of obesity. Genome-wide association analysis has revealed numerous genes related to obesity, but the genetic variation in these genes can only explain a fraction of the genetic risk for obesity [11,12]. In fact, the occurrence and development of obesity are largely influenced by environmental factors such as lifestyle, dietary habits, physical activity levels, psychological stress, diseases, and so on [8] (Figure 1). Epigenetics involves the regulation of the function and expression of host genes through various epigenetic modifications without changing the genomic DNA sequence. This process subsequently impacts the physiological and pathological processes of host cells and the entire organism, serving as a crucial mechanism through which environmental factors affect the expression of host genes [11]. This mechanism provides an important theoretical foundation for us to comprehend how environmental factors contribute to the onset and progression of diseases. DNA methylation, the most common epigenetic modification in mammals, and its abnormal changes have been proven to be closely linked to the development and advancement of obesity [13,14,15]. Research has revealed that alterations in DNA methylation patterns, induced by high-calorie diets and sedentary lifestyles, can affect the expression of genes involved in energy metabolism and fat storage, ultimately promoting the onset of obesity.

Furthermore, more and more studies have confirmed that there is a strong relationship between obesity and intestinal flora [16]. The intestinal microbiota can not only mediate obesity by regulating human energy metabolism, triggering chronic inflammation and affecting intestinal hormone secretion, but also actively participate in the metabolism of bile acids, short-chain fatty acids (SCAFs), and carbohydrates, eventually leading to obesity. Recent research has demonstrated a notable correlation between intestinal flora and epigenetic spectrum through DNA methylation genomics and intestinal flora sequencing. Specifically, the composition and abundance of intestinal flora have been found to be related to the DNA methylation status of promoters of genes related to lipid metabolism and inflammation [17]. Epigenetics is closely related to external factors such as diet, environment, and psychological stress, which directly regulate the epigenetic mechanism by affecting the gut microbiota. The metabolites generated by the intestinal flora are guaranteed to maintain regulatory functions. The interaction between intestinal flora and DNA methylation is essential in the regulation and development of obesity, highlighting the interconnectedness of these factors [18]. Therefore, investigating the interaction between DNA methylation and intestinal flora composition in obesity could offer innovative therapeutic strategies for prevention and management. This review focuses on exploring the potential role of gut microbiota and DNA methylation in the pathophysiology and etiology of obesity.

## 2. DNA Methylation and Obesity

### 2.1. Overview of DNA Methylation

**Figure 2:**
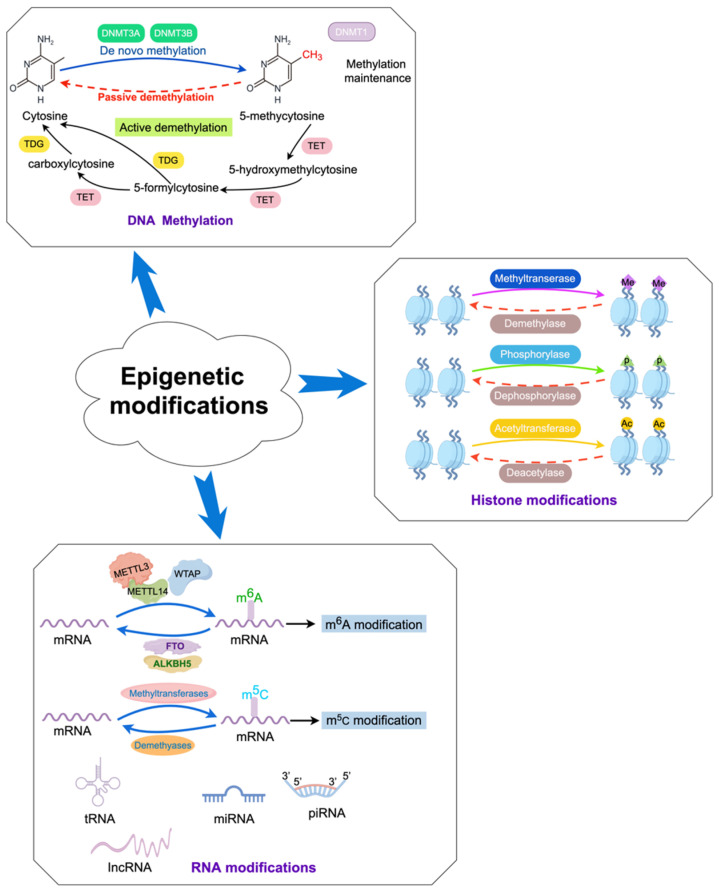
Dynamic epigenetic modifications in the host.

In mammals, DNA methylation refers to the process by which a methyl group (-CH_3_) is primarily added to the fifth carbon atom of cytosine in cytosine-phosphate-guanine (CpG) genomic DNA dinucleotides under the catalysis of DNA methyltransferases (DNMTs), forming 5-methylcytosine (Figure 2) [19,20]. S-adenosylmethionine is the main source of the methyl group (-CH_3_). DNA methylation can induce changes in chromatin structure, DNA conformation, and DNA–protein interactions, thereby regulating gene expression (Figure 2) [21,22,23]. DNA methylation exerts regulatory control over gene expression through multiple mechanisms. On the one hand, it can impede the binding of certain transcription factors (TFs) to gene promoters, thus weakening gene transcription [24]. On the other hand, TFs can recognize methylated DNA and collaborate with other TFs to reshape chromatin structure, ultimately activating transcription and modulating gene expression levels [25]. Methylated DNA can be recognized by methyl-binding proteins, which subsequently direct protein complexes possessing chromatin remodeling activity to a designated region within the genome, thereby repressing gene transcription and reducing or suppressing gene activity [26].

It is estimated that there are approximately 28 million CpG sites within the mammalian genome, of which about 70–80% are methylated in normal healthy cells [27]. Under normal circumstances, the CpG sites within promoter regions exhibit a tendency towards hypomethylation or unmethylation, while the CpG sites outside of promoter regions tend to be hypermethylated. The hypermethylation in the promoter regions frequently results in gene silencing or downregulation, while hypomethylation can activate or enhance gene expression [19,28]. Additionally, hypermethylation within the gene body regions often facilitates gene expression [29]. Widespread hypomethylation of non-promoter regions will compromise chromosome stability and disrupt normal gene transcription, ultimately contributing to the development of various diseases. More importantly, the dynamic and reversible process of DNA methylation in the body is regulated by DNA methyltransferases and demethylases. Dysregulation of DNA methylation can disrupt gene expression, impact biological pathways, and eventually lead to the onset of various diseases, including obesity, type 2 diabetes mellitus (T2DM), autoimmune diseases, tumors, cardiovascular diseases, and neurological disorders [30].

### 2.2. DNA Methylation in Obesity (Figure 3)

Obesity is characterized by a range of pathological mechanisms, including lipid metabolism disorders, oxidative stress, chronic low-grade inflammation, insulin resistance, endocrine disturbances, and abnormal nervous system regulation. DNA methylation is closely related to the development of obesity by affecting gene expression, then disrupting the regulatory balance between obesity-promoting genes (such as FTO and PPARγ) and anti-obesity genes (such as LEP, GLP-1R, and POMC) [31,32,33,34,35]. Genome-wide studies have found that the global DNA methylation patterns in the adipose tissue and blood of obese individuals exhibited hypomethylation [14,36]. Currently, more than 700 obesity-related genes have been identified, and the expression of some obesity-related genes is potentially regulated by differentially methylated CpG sites [37,38]. Studies have shown that for every 0.1% increase in the average DNA methylation level at the CpG loci detected in the promoter region of the SLC6A4 gene, BMI increased by 0.3 kg/m^2^, body weight increased by 0.16 kg, and waist circumference increased by 0.78 cm [39]. Moreover, compared with the offspring of normal-weight mothers, there are multiple differentially methylated CpG sites in the cord blood DNA of the offspring of obese mothers. Nikpay et al. conducted a genome-wide search for DNA methylation sites that lead to obesity and identified seven CpG sites that have a causal relationship with the risk of obesity, namely cg21178254 (CCNL1), cg06028605 (SLC5Al1), cg02814054 (MAST3), cg02814054 (POMC, ADCY3, and DNAJC27), and cg01884057 (POMC, ADCY3, and DNAJC27) [40].

**Figure 3:**
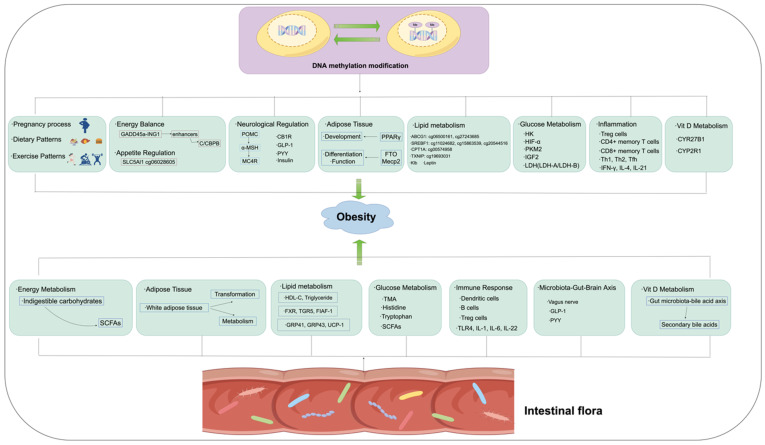
The possible mechanisms through which DNA methylation and intestinal flora contribute to the occurrence and development of obesity.

### 2.3. Role of DNA Methylation in Obesity

#### 2.3.1. DNA Methylation and Pregnancy, Dietary Patterns, and Exercise

The early stage of development is crucial for the creation and preservation of epigenetic markers. Research has found that when pregnant women experience extreme malnutritional deficiency or obesity, they increase the risk of obesity in their offspring in later life, which is possibly mediated by DNA methylation in the blood [41]. The nutritional level and habits of the mother during pregnancy can alter fetal development and subsequently lead to obesity in the future [41]. Proopiomelanocortin (POMC) protein is a precursor polypeptide that plays a role in regulating appetite and feeding behavior by producing a variety of active peptides, such as α-Melanocyte-Stimulating Hormone (MSH) [42]. Studies have found that a high-fat diet during pregnancy can lead to hypermethylation of the POMC promoter region, thus reducing the expression of POMC, weakening its functions of increasing satiety and suppressing appetite. This methylation state will persist in the offspring for a long time and may eventually lead to obesity and other metabolic issues [43].

Recent evidence suggests that dietary patterns strongly influence the epigenetic processes associated with the development of obesity. High-fat and high-sugar diets have been found to cause changes in DNA methylation patterns, contributing to diseases such as obesity and insulin resistance. A large intake of methyl-rich nutrients, for example, folic acid, vitamin B12, methionine, choline, etc., can affect the DNA methylation status of the host [43]. Certain specific ingredients in food, such as polyphenols (found in fruits, vegetables, and grains), catechins (found in green tea), etc. [44,45], can alter the DNA methylation status of some genes and play a role in improving obesity. An animal study indicated that a diet rich in methionine could promote atherosclerosis via the demethylation of fatty acid-binding protein (FABP). Therefore, a balanced diet and moderate intake of nutrient-rich foods are essential to maintain a healthy DNA methylation pattern [46].

As we all know, long-term persistent exercise, whether aerobic or anaerobic, contributes to burning energy and weight management, which is possibly related to the alteration of the DNA methylation patterns of some genes [47]. For instance, studies have found that long-term aerobic exercise is associated with decreased DNA methylation levels of some genes in blood and muscle cells. Conversely, high-intensity anaerobic exercise may lead to a significant increase in DNA methylation levels in some gene regions, possibly due to the physiological and oxidative stress induced by exercise affecting DNA methylation modifications [48,49].

#### 2.3.2. DNA Methylation and Energy Balance and Appetite Regulation

The imbalance between calorie intake and consumption is the fundamental pathophysiological basis of obesity. Excessive food intake leads to an energy surplus. At present, defective DNA methylation has been found in the promoter region of genes related to food intake and energy homeostasis in obese individuals. GADD45a-ING1 promotes enhancer demethylation to allow C/EBPB binding and transactivation of the target gene, thereby maintaining energy homeostasis [50]. The SLC5A11 protein is involved in the transport of glucose and other sugars, bile salts, and organic acids, and plays a role in regulating appetite. During starvation, the expression of SLC5Al1 increases and promotes the excitability of EB R4 neurons in SLC5Al1 by inhibiting dKCNQ activity, thus promoting feeding behavior. Hypomethylation of the cg06028605 site in the SLC5A11 gene results in obesity by reducing the expression of the gene [40].

#### 2.3.3. DNA Methylation and Neurological Regulation

The central nervous system (CNS) plays a pivotal role in regulating appetite, satiety, and energy balance. Various peptide hormones and signals from the gastrointestinal tract are released into circulation and play a role through transport into the central nervous system. Various satiety factors integrate signals into CNS pathways by interacting with vagus afferent fibers connected to higher brain centers, such as leptin, cholecystokinin, GLP-1, PYY, pancreatic polypeptide, etc. [51]. These central regulatory centers involved in regulating appetite and energy balance homeostasis integrate these signals, perceive the energy status, and give instructions. Subsequent neuronal efferent fibers and humoral outputs interact with peripheral target organs, thus controlling glucose metabolism, lipid metabolism, food intake, and energy homeostasis [52].

The arcuate nucleus complex (ARC), located near the central eminence of the hypothalamus, is a major energy balance regulation center in the CNS [53]. POMC neurons in the ARC nucleus primarily inhibit appetite by synthesizing and releasing POMC. Additionally, POMC is partially broken down into α-MSH. α-MSH acts on melanocortin-4 receptors (MC4Rs) in the hypothalamus and spinal cord to increase satiety and suppress appetite, therefore stimulating energy expenditure and reducing food intake, which is beneficial to reducing obesity. Conversely, agouti-related protein (AgRP) and neuropeptide Y (NPY) neurons in the ARC synthesize and release AgRP and NPY, which play a role in promoting appetite [54]. It has been found that abnormal hypomethylation of the Avy gene in agouti mice can lead to ectopic protein expression and binding to MC4Rs in the hypothalamus, which destroys the function of the MC4R and results in obesity. Interestingly, this phenotype was attenuated in the offspring of mice fed methyl-rich diets such as folic acid and methionine, confirming that DNA methylation is an important regulatory factor of obesity [55].

Circulating leptin is primarily secreted by adipose tissue and interacts with POMC and AgRP/NPY neurons. It binds to its receptor leptin regulatory protein (mainly expressed in hypothalamic neurons) to reduce appetite, increase satiety, and promote glucose homeostasis. This is helpful in controlling weight and glucose [51]. The cannabinoid neurotransmitter system influences central appetite control in the hypothalamic NPY and hypothalamic melanocortin feeding network. The cannabinoid-1 receptor (CB1R) is a crucial component of this system and is expressed in brain regions that affect mood, reward, appetite, and metabolism [56]. Glucagon-like peptide-1 (GLP-1) and peptide tyrosine-tyrosine (PYY) are hormones secreted by the intestinal tract. Cholecystokinin secretion is primarily influenced by food ingredients, and high-protein and high-fat foods can promote its release on a massive scale. After these hormone signals are transmitted to the satiety center, they all play an anorexic role and reduce food intake. Research has indicated that these genes are regulated by DNA methylation. Insulin, secreted by pancreatic *β*-cells after eating, acts as an anorexia signal when it enters the center, reducing food intake and body weight [57]. DNA methylation pattern is a key regulatory factor of genes related to pancreatic *β*-cell homeostasis, including insulin secretion and signal transduction [58].

#### 2.3.4. DNA Methylation and Adipose Tissue Development, Differentiation, and Function

It has been found that DNA methylation plays a crucial role in adipogenesis and the proliferation and differentiation of adipocytes. Inhibition of DNA methylation significantly suppresses adipogenesis in the early stage of 3T3-L1 preadipocyte differentiation, while inhibition of DNA methylation in the later stages of differentiation promotes adipogenesis [59]. It is suggested that DNA methylation plays a two-way regulatory role in adipogenesis. Peroxisome proliferator-activated receptor gamma (PPARγ) can activate the transcription of characteristic genes of adipocytes, which has been demonstrated to be a key regulator in the differentiation of preadipocytes into adipocytes and a necessary and sufficient condition for adipogenesis. Adipocytes treated with the DNA methylation inhibitor 5′-aza-cytidine can upregulate the expression of the PPAR-γ gene in a dose-dependent manner, thereby affecting adipose development [32]. The Fat mass and obesity-associated (FTO) gene is a key genetic predictor of obesity risk factors and is involved in the control of adipocyte differentiation [32]. Compared with the normal control group, the average expression level of the FTO gene in obese patients was higher. Knocking down FTO inhibited the differentiation of preadipocytes into adipocytes, while the overexpression of FTO enhanced this process. The effect of FTO on adipocyte differentiation is mediated through PPAR-γ, and the DNA methylation of FTO can promote inflammation by increasing the m^6^A RNA of PPAR-α [60].

Adipose tissue is classified into white adipose tissue (WAT), beige adipose tissue, and brown adipose tissue (BAT). WAT is the main part of the body used to store lipids, while BAT is a thermogenic tissue, which is more abundant in infancy. Beige adipose tissue is also a kind of thermogenic adipose tissue, its thermogenic activity is higher than BAT, and the total amount is more [61]. Adipose tissue regulates the energy status of the body through the secretion of and response to hormones. It was found that overall DNA methylation levels increased during the formation of WAT and BAT, with differentially hypermethylated CpGs mainly located in the promoter region and differences in DNA methylation levels between the two tissues [62]. Resistance training-induced reduction in the visceral fat area may be associated with increased DNA methylation of CpGs in the non-promoter region of FTO in whole blood [63]. These studies indicate the changes in DNA methylation during adipose tissue expansion and adipose formation. Previous studies have discovered that the “browning” of WAT is crucial in treating obesity. Methyl-CpG-binding protein 2 (Mecp2) is a chromatin-binding protein with a high affinity for methylated CpG DNA. The deletion of Mecp2 can promote fat browning and reduce obesity [64].

The segment is located in the promoter region of the FTO gene: 16:53703684–53703899. The methylation level of this important region in obese patients is significantly higher than in the normal group, and positively correlated with BMI, triglycerides, and waist circumference [31]. The methylation degree of the FTO CpG11 site was significantly reduced in infants with high birth weight [65]. An interesting study found a relationship between the methylation level of the FTO gene and its polymorphism in increasing the risk of obesity. Homozygous rs9939609AA individuals have a higher risk of obesity than heterozygous AT or homozygous TT individuals [66]. When the AA genotype is accompanied by enhanced methylation of the FTO gene, the risk of obesity is particularly increased. However, in the case of low FTO gene methylation, the difference between AA and TT homozygous variants was eliminated. Therefore, the methylation level is crucial for FTO gene polymorphism [66].

#### 2.3.5. DNA Methylation and Lipid Metabolism

An EWAS study on DNA methylation and lipid metabolism found that there was a relationship between multiple DNA methylation and the composition and concentration of lipids in different lipoprotein subclasses, including ABCG1 (cg06500161 and cg27243685) and SREBF1 (cg11024682, cg15863539, and cg20544516). The methylation level of these CpG loci was mostly positively correlated with lipid concentration, TG composition, HDL concentration, serum total TG, and MUFA measurements [67]. However, CPT1A (cg00574958) and TXNIP (cg19693031) exhibit opposite associations. The methylation of CpG sites in ABCG1, SREBF1, CPT1A, and TXNIP is mostly related to ApoB lipoproteins [67]. Fatty acids (FA) are mainly transported by ApoB lipoproteins and HDL. CPT1A is highly expressed in the liver, initiating the mitochondrial oxidation of long-chain fatty acids, thereby contributing to lowering serum TG levels [68]. The high DNA methylation regions of CPT1A are correlated with lower plasma total triglycerides and triglyceride-rich lipoproteins at baseline, such as cg00574958 [69,70]. ABCG1 is a gene encoding the protein for triglyceride metabolism, and its methylation is related to hypertriglyceridemia, glucose metabolism, and insulin sensitivity, which promotes obesity, T2DM, and hyperlipidemia. Functional enrichment analysis found that the methylation of ABCG1 methylation site cg06500161 may be associated with the significant lipid-lowering effects of lipid-lowering drugs and myocardial infarction related to the ABCG1 gene [71,72,73]. ELOVL2, a fatty acid elongase, is essential for the synthesis of polyunsaturated fatty acids (PUFAs) and is closely related to diabetes and aging. Its expression level in the liver is negatively correlated with DNA methylation. The DNA methylation of CpG islands in the Elovl2 gene (CGI-I1 in the first intron and CHI-E3 in the third exon) increased significantly with age in the liver [74].

The lack of DNMT inhibits the expression of genes involved in fatty acid oxidation and affects the expression of genes involved in fatty acid synthesis, which is related to the demethylation of the Klb promoter [75]. Beta-klotho (Klb) is an auxiliary receptor for the normal physiological functions of FGF15/19 and FGF21, which has been shown to counteract hepatic steatosis by coordinating the control of “stimulating fatty acid oxidation” and “inhibiting fatty acid synthesis” [76,77]. However, high-fat diets can enhance the expression of DNMT by increasing chromatin accessibility and reducing ubiquitin-mediated protein degradation, which leads to hypermethylation of the Klb promoter and subsequent downregulation of Klb expression, which in turn impairs fatty acid oxidation and oxidative phosphorylation, thus promoting abnormal accumulation of liver fat to denaturation in mice [74]. The hypermethylation level of DNMT3a and insulin-like growth factor 2 (IGF2) genes may increase the risk of metabolic syndrome in Chinese adults by affecting ELOVL6 activity dependence [77].

Leptin is one of the most extensively studied adipose factors secreted by adipocytes. In addition to regulating satiety centers, leptin also inhibits the production of fatty acid synthase, 3-hydroxy-3-methylglutaryl-CoA reductase, and glycerol-3-phosphate acyltransferase and upregulates the expression of adenylate-activated protein kinase to suppress lipogenesis and promote triglyceride and cholesterol breakdown and fatty acid oxidation. Studies have found that compared with normal children, leptin promoters in the peripheral blood, umbilical cord blood, and saliva of obese children showed hypomethylation, which is negatively correlated with body weight [78]. Kadakia et al. observed that methylation of five CpG sites in the leptin promoter in the blood of newborn infants is negatively correlated with maternal pre-pregnancy BMI. The study confirmed that methylation levels of the leptin gene promoter are decreased in obese women who have an effective response to calorie restriction.

#### 2.3.6. Association between DNA Methylation and Glucose Metabolism

Obesity is a common cause of insulin resistance, and insulin resistance can further exacerbate obesity [78]. MDB2, capable of recognizing DNA methylation, is a reader for deciphering the coding information of DNA and is also a susceptibility gene for obesity. Knocking out MBD2 in obese mice can effectively improve abnormal glucose tolerance and insulin resistance induced by a high-fat diet, thereby reducing obesity [79]. IGF2 competitively inhibits the binding between insulin receptors and insulin, disrupting normal insulin signal transduction and leading to insulin resistance. Compared with children born to non-obese parents, children born to obese parents exhibited abnormal methylation levels in the imprinting regulatory region of IGF2, and the instability of acquired imprinting may be transmitted to the next generation [80]. Glucokinase and pyruvate kinase play important roles in maintaining blood glucose homeostasis. Jiang et al. found that methylation levels of hepatic glucokinase and left pyruvate kinase were increased in obese rats fed with a high-fat diet, and were negatively correlated with gene expression, affecting glucose storage and glycogen synthesis and leading to elevated blood glucose [81].

Furthermore, DNA methylation can regulate glycolysis-related enzymes, inhibit mitochondrial function and glucose production-related enzymes, and promote aerobic glycolysis, which can meet the rapid energy demands of tumor cells. For example, the hypomethylation of the hexokinase (HK) promoter and the intron of pyruvate kinase M2 (PKM2) upregulates their expression, which ultimately promotes aerobic glycolysis in tumor cells, also known as the Warburg effect [82,83,84]. Lactate dehydrogenase (LDH) is a key enzyme in glycolysis. DNA methylation regulates the ratio of LDH-A/LDH-B by promoting hypermethylation in the LDH-B promoter region, then regulating the production of lactic acid [84]. Hypoxia-inducible factor (HIF) plays an important role in glycolysis. Interfering with DNA demethylating agents can significantly enhance the expression of HIF-α in colorectal cancer cells [85]. In addition, DNA methylation is also involved in regulating the degradation of HIF-α, and the hypermethylation of LIM domain-containing 1 [86,87].

#### 2.3.7. DNA Methylation and Inflammation

To a certain extent, obesity is considered a systemic subacute inflammatory state. Chronic low-grade local inflammation in adipose tissue and obesity-induced systemic inflammation may be the common factors driving obesity, in which T cells play an important role [88]. Treg cells are crucial immune cells for maintaining the balance of immune response in the body, while visceral adipose tissue (VAT)-Treg cells are lost under obese conditions [89]. Foxp3 is a key regulatory factor of Treg gene expression, and the demethylation status of the Treg cell-specific demethylated region (TSDR) seems to provide a basis for the epigenetic memory of Foxp3. Foxp3 CD44^−^hiCD4 memory T cells exhibit partially demethylated TSDR, which can express Foxp3 again after antigen stimulation, showing fully demethylated TSDR [90]. The differentiation of classic T-helper 1 (Th1) cells and Th2 cells is associated with the demethylation of interferon (IFN)-γ and interleukin (IL)-4 genes, respectively [91,92].

Additionally, DNA methylation influences immune memory responses, specifically in regulating the differentiation and effect functions of CD4^+^ memory T cells and CD8^+^ memory T cells. DNA methylation is very important for memory CD4^+^ T cells to retain their previous effector lineage after antigen clearance and to regain their lineage-specific effector function when they re-encounter antigens. For instance, the granzyme b (Gzmb) gene is unmethylated in the Th1 effector but is in a methylated state in Th1 memory cells. IL-21 and IFN-γ genes were demethylated in T follicular helper cells and Th1 cells, but remained unmethylated in memory T cells [93]. DNA methylation is important for controlling the expression of genes responsible for memory CD4/CD8 T cells, such as programmed cell death protein 1 (Pdcd1). The demethylation of Pdcd1 site occurs during the differentiation into functional memory CD8^+^ T cells.

#### 2.3.8. The Role of DNA Methylation in Vitamin D Metabolism

Vitamin (Vit) D is an endogenously synthesized hormone, and its active form, 1,25(OH)_2_D, regulates bone turnover and promotes various biological functions, including cell differentiation, proliferation, and immune system regulation [94]. As has been reported, Vit D has roles in inhibiting monocyte chemotactic protein and recruiting macrophages in adipose tissue. Studies have shown that obesity can disrupt the homeostasis of Vit D metabolism-related genes (such as CYR27B1 and CYP2R1) through abnormal DNA methylation, leading to disturbances in Vit D-related metabolites [95,96]. In turn, lower levels of Vit D will exacerbate the progression of obesity through various mechanisms. It has been found that the decreased expression of Vit D in the blood of obese patients promoted obesity by affecting insulin resistance, endocannabinoid activity, glucose and lipid metabolism, chronic inflammation, and adipocyte differentiation. Hypermethylation levels in the promoter regions of Vit D metabolic pathway genes may lead to gene silencing and affect Vit D changes.

Vit D is generally believed to regulate inflammatory responses by modulating the production of inflammatory cytokines [97]. Studies have found that a higher degree of Vit D deficiency is associated with lower levels of DNA methylation, inducing the expression of inflammatory adipokines, such as C-X-C motif chemokine ligand 8 (CXCL8), IL-12A, and NF-κB, thus inducing local chronic inflammation. In addition, Vit D has a strong positive correlation with the DNA methylation of inflammatory adipokines in the adipose tissue of obese individuals, including TNF-α, CRP, chemokines, and adhesion molecules [96]. Supplementation of Vit D can reduce CRP and TNF-α in obese diabetic patients and lead to a shift in T cell phenotypes from pro-inflammatory (Th1/Th17) to anti-inflammatory (Th2/Treg) [98].

## 3. Intestinal Microbiota and Obesity (Figure 3)

### 3.1. Overview of Intestinal Microbiota

The intestinal microbiota, i.e., the gut microbiome, consists of approximately 100 trillion kinds of bacteria, including bacteria, archaea, viruses, and eukaryotes, colonizing the human digestive tract. The gut microbiota encodes around 3.3 million specific genes, producing a variety of metabolites that participate in the biochemical pathways of the host [99,100]. At the phylum level, the predominant phyla are *Firmicutes* (accounting for 60%), *Bacteroidetes*, and *Actinobacteria*, and at the genus level, *Pseudomonas*, *Streptococcus*, and *Prevotella* are the main genera. Based on the PH gradients and oxygen levels, each flora inhabits different parts of the gastrointestinal tract [101].

Newborns acquire their microbial communities from the environment during delivery, or from their mothers through breastfeeding, gradually maturing thereafter [102]. Compared with the infants born via cesarean section, the intestinal flora in the newborns born via natural delivery is primarily composed of *Lactobacilli*, *Bacteroides*, and *Prevotella*, while in the former, it mainly consists of *Clostridium*, *Clostridiales*, and *Escherichia coli* [103,104,105,106,107]. Additionally, compared with those fed with infant formula, the gut microbiota of breast-fed newborns was mainly dominated by *Bifidobacterium*, *Lactobacillus*, *Staphylococcus*, and *Streptococcus* [105,108]. The microbial community experiences significant changes during infancy and then transitions to a more mature pattern at around 3 or 4 years of age, after which the change rate of the species composition of intestinal flora slows down. Until adulthood, in the absence of major environmental disturbances, the microbiota tends to be stable, forming the core intestinal flora [109].

The core intestinal flora plays a crucial role in maintaining human health. It influences various physiological and metabolic processes of the human body through its partial structure and the microbial metabolites, including digestion, nutritional absorption, regulation of intestinal hormone secretion, modulation of intestinal immunity and inflammatory processes, synthesis of vitamins, amino acids, and various metabolites (such as SCFAs, choline, and lipids), and regulation of host gene expression [110,111,112]. In healthy individuals, most of the gut microbiota are in a state of dynamic balance. When the interference of diet, living environment, psychological stress, infection, the use of antibiotics, and other factors leads to significant changes in the species and abundance of intestinal microorganisms, it is called intestinal flora imbalance [113]. Dysbiosis of the gut microbiota has been proven to be related to the increased incidence of many diseases, including metabolic disorders (such as T2DM and obesity), atherosclerosis, stroke, autoimmune diseases, cancer, etc. [111,114,115,116,117].

### 3.2. Role of Intestinal Flora in Obesity

It is indicated that the intestinal flora of obese individuals is in a state of dysbiosis, which is characterized by a decrease in biodiversity [118], a decrease in microbial gene richness, an imbalance in the *p_Firmicutes*/*p_Bacteroidetes* ratio [119], an increase in bacterial *Proteobacteria*, and a decrease in *Clostridium*, among other factors. The phylum *Bacteroidetes* is associated with the intake of energy and fatty acids. In general, individuals with obesity show an increase in the phylum *Firmicutes* and a decrease in *Bacteroidetes*, resulting in an imbalance between the two [114], which can be improved through dietary interventions [114,118]. Moreover, the numbers of beneficial bacteria, such as *Lactobacillus*, *Bifidobacterium*, and *Clostridium*, may be reduced in individuals with obesity, while the numbers of some harmful bacteria, such as *Proteobacteria*, may increase [120,121], contributing to flora imbalance. Following diet interventions, there is an increase in intestinal microflora diversity and gene richness, and their composition will shift to the composition of lean individuals [114,118]. Therefore, microbial imbalance may be one of the main factors contributing to obesity. *A.muciniphila* is associated with the restoration of intestinal barrier function and the reduction of endotoxemia [122]. In obese mice, oral administration of *Akkermansia* muciniphila could reduce fat gain and adipose tissue inflammation, while enhancing intestinal barrier function. Prebiotics play a role in animal models of obesity and diabetes by promoting the proliferation of beneficial bacteria such as *Lactobacillus* and *Bifidobacterium*, improving enteroendocrine cell activity, enhancing the postprandial secretion of hormones that promote satiety, improving glucose and lipid metabolism, increasing leptin sensitivity, and improving intestinal barrier permeability [123,124,125]. In recent years, fecal microbiota transplantation and prebiotics have been used in the treatment of obesity and have achieved good therapeutic effects [126,127].

Indeed, the gut microbiota does affect body weight. Germ-free mice showed lower body weight and white adipose tissue content compared to conventional mice, but after receiving a transplant of normal intestinal microbiota (IM), their body weight was similar to conventional mice within two weeks [128]. The body weight of mice fed with a high-fat diet was significantly higher than that of mice fed with a low-fat diet, whereas the body weight of germ-free mice remained stable under the two feeding methods, suggesting that IM contributes to correctly dealing with energy storage from food intake [129]. Furthermore, the increase in body weight of germ-free mice depends on the source of fecal microbiota transplantation (FMT). Compared with FMT from lean mice, even when fed the same diet, FMT from obese conventional mice to germ-free mice leads to higher weight gain and white adipose tissue reserves [130,131,132]. Lastly, FMT from obese individuals into germ-free mice resulted in higher weight gain than their lean twin counterparts [133].

The potential mechanisms of obesity caused by intestinal microflora may include the following: (1) fermenting dietary carbohydrates that are difficult for the host to digest into short-chain fatty acids (SCFAs), thereby helping the host to obtain additional energy [134]. (2) affecting the metabolism, conversion, and reabsorption of bile acid in the host, thereby regulating the composition of bile acid and affecting its biological function in lipid metabolism, insulin sensitivity, immunity, and so on [135]. (3) regulating host appetite and eating behavior through the microbiota–gut–brain axis, involving the brain, immune, and hormonal regulatory systems between the gut and the brain, which can activate gastrointestinal hormones such as G protein-coupled receptors and gastrointestinal peptides, which in turn affect host appetite and eating behavior [136]. (4) shaping the immune system and inducing inflammation. After the intestinal mucosal barrier function was damaged after the imbalance of flora, endotoxin LPS was absorbed into the blood through the intestine and induced inflammation [137,138]. (5) participating in the synthesis of host vitamins, such as Vit D and Vit E [139]. (6) participating in epigenetic modifications of host genes, such as DNA methylation and histone modifications, etc. [117]. Next, we will elaborate on the metabolites of intestinal flora to explain in detail the mechanism involved in the occurrence and development of obesity.

#### 3.2.1. The Role of Intestinal Microbiota in Energy Metabolism

The intestinal microbiota produces relevant enzymes to break down indigestible carbohydrates, such as dietary fibers, into SCFAs including acetic acid, propionic acid, and butyric acid. These SCFAs are then provided to the host for application or storage as primary energy [140]. Compared with conventional mice, the digestion and absorption capacity of germ-free (GF) mice fed a high-fat diet was reduced, resulting in an increase in 24 h fecal volume and calorific content in feces [141,142]. Moreover, this diminished digestion and absorption capacity is the common result of the interaction between gut microbiota and host lipids. The feces of GF mice contain 40% more lipids, including total cholesterol and triglycerides. This difference in energy extraction from food is partly due to the functional characteristics of intestinal microbiota [141,142]. Additionally, compared with thin mice, the cecal intestinal microbiota of obese mice showed a higher concentration of enzymes that break down indigestible carbohydrates, leading to an increase in SCFA production and consequently providing more energy for the body [132,143]. Fasting-induced adipose factor (FIAF) not only affects adipose tissue metabolism by inhibiting fatty acid uptake and synthesis but also regulates blood glucose levels by regulating the insulin signal transduction pathway in the liver. Intestinal microbiota can inhibit FIAF expression, enhance the activity of lipoprotein lipase, and promote lipid storage in WAT. These findings emphasize the connection between intestinal microbiota, the gut tract, and energy acquisition and storage [144,145]. Gut microbiota may regulate obesity by modulating the expression of LPL inhibitor FIAF expression, which influences energy extraction and distribution.

#### 3.2.2. The Role of Intestinal Microbiota in the Transformation and Metabolism of White Adipose Tissue

Metabolites produced by gut microbiota, such as SCFAs, upon reaching adipose tissue through the bloodstream, activate G protein-coupled receptors on the surface of adipocytes, promote the differentiation of preadipocytes into mature adipocytes, and increase the storage capacity of adipose tissue [146]. Of special concern is that gut microbiota can also influence the “browning” of adipose tissue. WAT “beiging” refers to the process in which beige fat cells appear in white adipose tissue, exhibiting characteristics similar to brown adipose tissue. Beige cells can generate heat because of their higher mitochondrial content and expression of characteristic brown fat markers such as uncoupling protein 1 (UCP1) [147]. This process is usually induced by external stimuli (such as cold exposure and exercise) or internal factors. Increasing the number and activity of beige cells can enhance energy expenditure, improve insulin sensitivity, and promote fatty acid metabolism. Studies have shown that compared with mice exposed to room temperature, mice exposed to cold temperatures consumed more calories, but experienced weight loss accompanied by changes in their intestinal microbiota composition. After the fecal microbiota of these two groups of mice was transplanted into conventional mice, it was found that the mice that received FMT from mice in a low-temperature environment showed a decrease in body weight and fat mass, an improvement in insulin sensitivity, an increase in energy consumption, the formation of WAT beiging, and an increase in UCP1 expression in WAT [148,149]. These data indicate that intestinal microbiota is involved in adipose tissue differentiation and the beiging effect in WAT.

Some metabolites produced by microbes can regulate the expression of microRNAs (miRNAs) in WAT, such as the miR-181 family, which in turn can regulate host energy consumption and body weight. miR-181 participates in metabolic fitness by controlling the expression of genes involved in metabolic adaptability, adipocyte function, and insulin signal transduction [150]. In the WAT of diet-induced obese mice and obese individuals, the expression of miR-18 was significantly upregulated. In contrast, miR-181-KO mice fed an HFD did not develop obesity compared to WT mice, and they showed decreased WAT, smaller adipocyte volume, and increased energy consumption [151]. Furthermore, it was found that the expression of miR-181 in the WAT of germ-free mice was lower than that of conventional mice, and transplantation of fecal microbiota from conventional mice into germ-free mice could lead to an increase in miR-181 in WAT, indicating that intestinal microbiota regulates the expression of miR-181 [150,151]. Indole, as a metabolite of microbial breakdown of tryptophan in the gut, can reduce the expression of miR-181 in WAT and prevent diet-induced obesity [151]. These findings demonstrate the inter-conductance between intestinal microbiota and its metabolites and micro-RNA in WAT plays a necessary role in controlling weight.

#### 3.2.3. Functional Mechanisms of Intestinal Microbiota in Lipid Metabolism

Lipidomic analysis revealed that the levels of systemic triglycerides, total cholesterol, and high-density lipoprotein cholesterol (HDL-C) were elevated in germ-free mice compared to conventional mice, while hepatic cholesterol was increased [152]. In comparison to conventionally raised ApoE^−/−^ mice, ApoE^−/−^ mice were fed with broad-spectrum antibiotics, increasing their cholesterol levels, particularly very-low-density lipoprotein (VLDL) and low-density lipoprotein cholesterol (LDL-C), indicating that IM affects the lipid profiles of mice and is modulated by genetic factors [153]. Studies have shown that the composition of gut microbiota explains 6% and 4% of the alteration in triglycerides and high HDL-C levels, respectively, with almost no significant impact on LDL-C levels [154]. This suggests that gut microbiota can alter the composition of lipids in the blood.

Bile acid, acting as an emulsifier, facilitates the digestion and absorption of lipids by making lipid molecules more accessible to digestive enzymes in aqueous solutions. As a microbial metabolite, it can regulate the expression of lipid transporters in intestinal cells, such as NPC1L1 and ACBG5/ABCG8, by binding to receptors, such as farnesoid X receptor (FXR) and Takeda G protein-coupled receptor 5 (TGR5), on the surface of intestinal cells, modulating lipid absorption [155,156]. Enterohepatic circulation ensures a continuous supply of bile acid. The gut microbiota, especially genera *Clostridium*, *Bacteroides*, *Parabacteroides*, and *Turicibacter*, can convert primary bile acid into secondary bile acid through enzymes, which can be used for bile acid resynthesis [157,158,159,160]. Therefore, intestinal microbiota increases the diversity of bile acids and is conducive to the role of various bile acids. Additionally, the gut microbiota contributes to bile acid reabsorption by altering intestinal PH value and producing bile salt-binding proteins. Imbalance of gut microbiota leads to intestinal mucosal barrier disorders, which will damage bile acid circulation, affecting lipid transport, digestion, and absorption.

FIAF promotes the catabolism of fatty acids by increasing the activity of AMP-activated kinase in the colon, liver, and skeletal muscles. It also inhibits the activity of lipoprotein lipase and fat production, exerting a protective effect against obesity. However, transplantation of intestinal microbial communities inhibited the expression of FIAF in intestinal epithelial cells and increased fatty acid uptake by adipocytes, and therapy promoted fat storage. It was found that the lack of intestinal flora in germ-free mice on a high-fat diet exhibited increased FIAF expression, which inhibited the activity of lipoprotein lipase (LPL), resulting in an increase in postprandial triglyceride concentrations [161,162]. After treatment with a low-fat diet (LFD) and LPL inhibitors, the absorption of triglyceride and cholesterol in germ-free mice decreased compared with conventional mice. Moreover, a high-fat diet fails to restore the systemic lipid levels of germ-free mice after LFD, which demonstrates the importance of IM in lipid absorption [142]. By transplanting IM from conventional mice fed an HFD into germ-free mice, lipid absorption in germ-free mice was restored to the same extent as in conventional mice [142]. Additionally, an HFD induced some IM (such as *Bifidobacteria* and *Lactobacilli*) to increase the expression of acylglycerol o-acyltransferase (triglyceride synthase) [142,163,164].

Microbially derived acetate participates in the de novo synthesis of fatty acids, especially SCFAs, while mice treated with antibiotics showed a reduction in fatty acid synthesis [152]. SCFAs induced the activation of G protein-coupled receptors 41 (GRP41) and GRP43, and the binding of SCFA and GRP43 promotes lipogenesis [165,166]. Butyrate appears to enhance fatty acid oxidation and thermogenesis by increasing the expression of PPAR-γ coactivator 1-alpha and mitochondrial uncoupling protein 1(UCP-1) in brown adipose tissue, as well as the phosphorylation of AMP-activated kinase in muscles and the liver [167]. Butyryl coenzyme-α-acetate-coenzyme-α transferase is an important enzyme in the production of butyrate in IM. A human study has confirmed a negative correlation between circulating triglyceride levels and the enzyme, further establishing the link between IM, SCFAs, and lipid concentrations in the human body [168].

#### 3.2.4. The Functional Mechanisms of Intestinal Microbiota in Glucose Metabolism and Insulin Resistance

The development of insulin resistance is formed by the complex effects of different metabolites affecting insulin signaling and inflammation. Several metabolites derived from intestinal microbiota (IM), such as branched-chain amino acids and their metabolites, tryptophan and its metabolites, SCFAs, etc., are involved in the occurrence and development of obesity by affecting insulin resistance [169]. Trimethylamine (TMA) is produced by intestinal microbiota from choline, L-carnitine, cholesterol, and other choline-containing compounds, then converted to trimethylamine-N-oxide (TMAO) in the liver [170]. TMAO has been found to affect insulin signal transduction and promote insulin resistance [171,172,173]. In addition, endotoxins associated with intestinal microbiota enter the blood into various tissues through the intestinal mucosa, causing chronic low-grade inflammation, which is one of the important mechanisms for the development of insulin resistance. *Alloprevotella* and *Allobaculum*, producers of SCFAs, are associated with improvements in obesity and insulin resistance [174].

IM degrades histidine to produce indole propionic acid. Compared with healthy individuals or patients with impaired glucose tolerance, indole propionic acid was increased in patients with diabetes. However, injections of indole propionic acid in mice can increase fasting and postprandial blood glucose levels by impairing insulin signaling [175]. Tryptophan is an important amino acid in the human body, and IM can decompose it into indole and its derivatives, which regulate insulin release and glucagon-like peptide 1 (GLP-1) production by binding to the aryl hydrocarbon receptor (AhR) [176]. It has been found that during obesity, intestinal inflammation increased, indole decreased in feces, and AhR activity decreased [177]. However, AhR agonist therapy can improve glucose tolerance, insulin signaling, and HFD-induced inflammation in the intestine and white adipose tissue in mice [178]. In addition, GLP-1 production promoted by indole metabolites not only affects the release of insulin but also acts on satiety [179]. Tryptophan is also metabolized to kynurenine by the rate-limiting enzyme Indoleamine 2,3-dioxygenase 1 (IDO1) [176]. IDO1 was activated in obese individuals and HFD-induced obese mice, leading to increased kynurenine [180]. IDO1 deficiency in mice fed an HFD can prevent obesity, WAT inflammation, liver steatosis, and insulin resistance, but this change is not observed after treatment with antibiotics, suggesting that IM plays a certain role in this process [181].

Intestinal microbiota produces three main SCFAs, butyrate, propionate, and acetate, during dietary fiber fermentation. It is known that acetate and propionate are involved in fundamental metabolic processes, such as gluconeogenesis and adipogenesis in the liver. Intestinal cells secrete GLP1 and PYY when these SCFAs are combined with GRP41 and GRP43, thereby improving insulin sensitivity [182,183,184,185]. It has been demonstrated that glucose tolerance, insulin resistance, and beneficial metabolites (such as succinate) can be improved by altering the composition of the intestinal microbiota to elevate GLP-1 levels [186]. Furthermore, it is also shown that SCFAs regulate glucose metabolism through intestinal gluconeogenesis [186]. Butyrate is an essential energy substrate for colonic mucosal cells and has a positive effect on insulin sensitivity. Oral butyrate can improve the insulin sensitivity of HFD-fed mice and reduce body weight by increasing energy expenditure [167].

#### 3.2.5. The Role of Intestinal Microbiota in Immune Response

In the early stages of life, the colonization of intestinal microbiota is crucial for the maturation of the immune system [187]. The initially established intestinal flora of newborns promotes the establishment of immune tolerance by interacting with immune cells, such as dendritic cells and B cells [188]. Metabolites of intestinal microbiota, such as SCFAs, can be used as signal molecules to influence the differentiation of regulatory T cells (Treg), the balance of Th17/Treg cells, and regulate the balance of autoimmunity [189,190]. In addition, metabolites of intestinal microbiota can also be used as chemical chemokines to induce the migration of dendritic cells (with antigen presentation) and affect the activation of T cells and B cells [191]. In immune cells, the binding of SCFAs to GPRs may alleviate the progression of inflammation. More importantly, intestinal microbes protect the host from pathogens by producing pro-inflammatory cytokines such as IL-1 and IL-6 [192].

Research has shown that a decrease in microbial gene richness is associated with low-grade inflammation, while the diversity and richness of intestinal microbiota in obese individuals are significantly decreased, which indirectly indicates that changes in intestinal microbiota may mediate the inflammatory response of obesity. Indeed, intestinal inflammation increases during obesity and is negatively correlated with the expression of AhR and IL-22 genes [193], whereas treatment with AhR agonists restores the tight junctions of intestinal epithelial damaged by palm oil feeding and improves intestinal inflammation [193]. Changes in intestinal microbiota may reduce the integrity of the intestinal mucosal barrier, leading to increased leakage of the endotoxin LPS and fatty acids. After LPS binds to TOLL-like receptor 4 (TLR4), it activates the early pro-inflammatory responses associated with obesity [194]. The leakage of fatty acids will further trigger the endoplasmic reticulum stress response, which exacerbates the inflammation induced by TLR4 [195,196]. IM regulates the expression of many hepatic genes, especially those that transport LPS via Myd88 signals [194].

#### 3.2.6. The Role of the Microbiota–Gut–Brain Axis in Obesity

The Microbiota–Gut–Brain Axis (MGBA) refers to the complex network of interactions between the gut microbiota and the brain, involving signaling transduction between the intestinal tract, nervous system, and endocrine system [155]. The MGBA plays a more and more important role as a central regulator of metabolism and appetite. Appetite control pathways include enteroendocrine cells (EECs), the vagus nerve (VN), gut hormones, the hypothalamic appetite center, and so on [197]. The VN represents a potential pathway for gut microbiota to influence host feeding behavior [197]. The MGBA plays a key pathophysiological role in the development of obesity and metabolic disorders through the vagus nerve and GLP-1 of intestinal microflora [136]. In the large intestine, the gut microbiota utilizes undigested dietary nutrients, fibers, and bile acid to produce metabolites such as SCFAs and indole, thereby inducing enteroendocrine L cells to secrete GLP-1 [198]. In the distal intestine, the release of GLP-1 and PYY is predominantly activated by the gut microbiota through their membrane components, such as lipopolysaccharides or metabolites, thus maintaining the secretion of GLP-1 and PYY within several hours after a meal. These gastrointestinal hormones can be fed back to the central nervous system via the gut–brain axis to regulate satiety and appetite [155,199].

SCFAs may contribute to the control of energy intake and utilization via the gut–brain axis [136]. Alterations in gut microbiota can also change the production of gastrointestinal peptides associated with satiety, leading to an increase in food intake. The dysbiosis of the gut microbiota due to an imbalanced diet may lead to changes in neurotransmitters, resulting in overeating and increased weight gain. Regulating gut hormone release through the application of synchronized appetite/satiety signals and energy balance mechanisms is a potential therapeutic target for obesity.

#### 3.2.7. Intestinal Microbiota and Vitamin D Metabolism

Bile acid, derived from cholesterol, is primarily synthesized by the liver and transported to the intestine tract, then chemically modified twice under the action of intestinal microbiota to form secondary bile acids with increased lipophilicity. Most bile acids are reabsorbed by the liver through the enterohepatic circulation. The most abundant metabolites in the gut microbiota, including secondary bile acids, deoxycholic acid, and lithocholic acid, have been shown to regulate host energy homeostasis and promote metabolism by activating TGR5 [200,201]. Bile acid metabolism plays a significant regulatory role in obesity, metabolic syndrome, and other diseases. Recent studies have found that the gut microbiota–bile acid axis partly mediates the association between plasma 25(OH)D levels and the risk of persistent Metabolic Syndrome (MetS) and MetS events in the Chinese population [202]. A decrease in secondary bile acids and other microbial metabolites results in a decrease in Vit D concentration, which was significantly correlated with an increase in *Turicibacter* abundance. It is speculated that the gut microbiota *Turicibacter* may mediate the decline of Vit D in obesity. A higher Vit D concentration was positively associated with higher alpha diversity of the gut microbiota and negatively associated with metabolic syndrome. Compared to the low-Vit D group, the high-Vit D group was enriched in *Ruminiclostridium-6* and *Christensenellaceae R-7*, which are negatively correlated with MetS, while *Lachnoclostridium* and *Acidaminococcus* showed the opposite correlation [202]. Therefore, the gut microbiota may mediate the decrease in Vit D and trigger the immune response and calcium absorption, thereby promoting the occurrence of obesity and metabolic syndrome.

## 4. Interactions between Intestinal Microbiota and DNA Methylation

DNA methylation and gut microbiota homeostasis are dynamic processes largely regulated by environment and diet. This suggests that they may share common triggers and jointly regulate the pathophysiology of diseases. With the development of omics technologies, it has been discovered that there is indeed crosstalk between DNA methylation and gut microbiota, and their interaction plays a multifaceted role in maintaining host health and preventing disease [203]. It has been found that anthocyanin, a polyphenol in black raspberries, could downregulate the expression of DNMT3A, DNMT3B, and p-STAT3 in CRC, leading to demethylation of the SFRP2 gene promoter and increasing the expression of SERP2 at mRNA and protein levels [204,205]. Along with this epigenetic change, the microbial structure also undergoes alteration, implying an internal correlation between DNA methylation and the gut microbiota. However, at present, the exact mechanisms underlying the interaction between gut microbiota and DNA methylation modification remain largely unknown.

### 4.1. The Impact of Intestinal Microbiota on DNA Methylation

Previous research has discovered that the intestinal microbiota has widespread influences on the DNA methylation of multiple cell types and tissues in mammals. After treating IECs with probiotics and pathogenic bacteria, respectively, DNA methylation modification was initiated in more than 200 methylation regions. The analysis of transcriptome and global DNA methylation of colonic epithelial cells from germ-free and conventionally housed (CNV) mice revealed that exposure to microbiota induced DNA hypomethylation and increased the expression of antibacterial and anti-inflammatory genes, thus promoting the metabolic homeostasis of these animals [206]. However, this microbiota-dependent DNA demethylation corresponds to increased expression and activity of the DNA demethylase TET3 and methyltransferase DNMT1 in the colon epithelium of CNV mice [206,207]. The deletion of IEC-specific TET leads to an increase in global DNA methylation, including sensitive areas related to microbial community [207]. The elevated level of *Fusobacterium* was related to the increased DNA methylation of colorectal cancer-related genes in patients with ulcerative colitis. When microbiota related to colorectal cancer was transplanted into germ-free mice, intestinal epithelial proliferation and DNA methylation were promoted [208,209].

A recent clinical study on metabolic syndrome has shown that fecal microbial transplantation (FMT) can not only modulate the composition of gut microbiota and plasma metabolites but also affect DNA methylation patterns in peripheral blood mononuclear cells. After 6 weeks of FMT, there was a significant difference in DNA methylation sites between autologous and allogeneic FMT groups, which was associated with insulin sensitivity [210,211]. Germ-free (GF) mice without intestinal microbiota showed a significant reduction in the degree of intestinal DNA methylation compared with CNV mice [212]. In the colonic epithelial cells of GF mice, decreased DNA methylation was observed in the 5ʹ CpG island region of the TLR4 gene, resulting in reduced TLR4 expression. Furthermore, the CXCL16 gene in the colon and lung of GM mice exhibited demethylation, which was consistent with the decreased accumulation of mucosal resident natural killer T cells in the absence of microbiota [213]. Scholars believe that the root cause of this DNA hypomethylation should be the reduction of one-carbon metabolites derived from the intestinal microbiota [214]. Uhrf1 is a DNA methylation adapter protein that forms a complex with DNMT1 and HDAC1. T cell-specific deletion of Uhrf1 will affect the expression of normal cell cycle genes in Treg cells, leading to spontaneous colitis. Moreover, the intestinal microbiota regulates the DNA methylation of Treg cells via promoting Uhrf1 expression, thereby modulating the occurrence of spontaneous colitis [215]. Toll-like receptor 2 (TLR2)-knockout mice presented hypermethylated promoter regions of ANPEP and IFIT2 genes, which are involved in the immune process. Most importantly, the epigenomic and transcriptomic modifications were linked with the alterations in the microbial composition of their colon mucosa, including significant changes in the abundance of *Firmicutes* [216]. This indicates that TLR2 may indirectly affect the DNA methylation status of some genes in the host by influencing the microbial composition of the colon mucosa. In summary, these studies have relatively directly shown that intestinal flora could affect DNA methylation modification in the host, thereby impacting the occurrence and development of diseases.

The gut flora, as an environmental factor, could affect the DNA methylation modification of the host under the influence of diet, drugs (such as antibiotics), and other factors. Currently, it is believed that the intestinal flora mainly affects the DNA methylation pattern of the host through its derived microbial metabolites [217]. The involved specific mechanisms include: (1) providing a chemical donor, a methyl group for DNA methylation, and (2) regulating the expression and/or activity of enzymes participating in DNA methylation modification.

#### 4.1.1. Intestinal Flora Provides Methyl Group (-CH_3_) for DNA Methylation

DNA methylation is a modification process, mediated by DNA methyltransferases (DNMTs), in which a cytosine in the genome covalently binds a free methyl group. DNMTs usually rely on methyl donors to catalyze their activity. Thus, the supply of methyl donors is a crucial step in DNA methylation modification. Although methyl donors could be generated through host-intrinsic pathways, increasing evidence suggests that the gut microbiota may serve as an additional source of methyl donors. In fact, the intestinal microbiota is capable of synthesizing various biological metabolites, including substrates and cofactors of DNA methylation, and regulators of DNA methyltransferase activity [218,219].

S-adenosylmethionine (SMA) is an important methyl donor, and folic acid, vitamin B2, vitamin B6, vitamin B12, methionine, and choline are important sources for the synthesis of SMA [45,220], which could provide essential methyl groups for DNA methylation processes. The intestinal flora is directly able to participate in regulating host DNA methylation modification by synthesizing these methyl sources. For instance, *Bifidobacterium* and *Lactobacillus* can not only synthesize folic acid [221] but also directly metabolize dietary methionine into SAM. *Latilactobacillus sakei LZ217* also showed the potential to produce large amounts of folic acid. Polyamines are rich in methyl groups and could be used as methyl donors, while the high levels of polyamines and choline in the intestine could be produced by the gut microflora. The intestinal flora, *Bifidobacterium*, *Lactobacillus*, *Bacteroidetes*, *Fusobacteria*, and *Proteobacteria*, also participate in the synthesis of B vitamins [221], including vitamin B2, vitamin B6, and vitamin B12, which have an important influence on the methylation status of host immune cells. For example, vitamin B12 deficiency in reproductive-age women resulted in hypomethylation of the promoter regions of two key genes, SREBF1 and LDLR, involved in cholesterol biosynthesis, causing increased cholesterol synthesis rates [222]. Vitamin B2 promotes increased methylation of the ZAC1 gene, which is involved in fetal growth and metabolism [223]. Dysbiosis of the intestinal microflora will affect the concentration of these vitamins. Therefore, the change in gut flora composition may affect methyl donor levels, thereby influencing the DNA methylation status of the host.

#### 4.1.2. Intestinal Flora Participates in the Expression and/or Activity of DNA Methylation Modification Enzymes

In mammals, DNMTs are divided into two types according to differences in structure and function: maintenance DNA methyltransferase (DNMT1) and de novo DNA methyltransferase (DNMT3A, DNMT3B, and DNMT3L). The DNMT1 family replicates the existing methylation pattern to the new DNA strand in the process of DNA replication to maintain the DNA methylation state. DNMT3A and DNAMT3B not only bind unmethylated DNA in the early development process but also establish new DNA methylation markers in differentiated cells in a signal-dependent manner. DNMT3L increases the binding of other DNMT3 to methyl donors. However, the newly discovered DNMT2 methyltransferase is rarely involved in the setting of DNA methylation patterns [224]. Studies have shown that intestinal flora could activate DNMA1 and regulate the methylation of 3′CpG islands (CGIS) that contribute to the maturation of intestinal epithelial cells (IECs) and participate in the epigenome of IECs [225]. It is suggested that intestinal flora may affect the expression of DNA methylase through its derived metabolites.

Short-chain fatty acids (SCFAs) are important molecules related to epigenetics, including acetate, propionate, butyrate, succinate, and lactate, which are produced by the fermentation of indigestible carbohydrates and fibers by intestinal microorganisms [226]. It was found that the SCFA butyrate can downregulate the level of DNMT1 by inhibiting the phosphorylation of MAP kinase 1 (ERK) [227]. The gut microbiota, including microbes such as *Coprococcus*, *Porphyromonas*, *Faecalibacterium*, and *Anaerotruncus*, serves as an important source of butyrate [228]. Therefore, the gut microbiota could inhibit DNMT activity by participating in the synthesis of butyrate and other SCFAs, ultimately affecting DNA methylation. Polyphenols are widely distributed in fruits, vegetables, and plants, and are also produced through intestinal microbial metabolism, e.g., by *Bacteroides*, *Clostridium*, *Eubacterium limosum*, and *Eggerthella lenta*, which are converted into various aromatic SCFA derivatives in the intestine, such as phenylacetate or phenylbutyrate [229]. Polyphenols have a wide range of activities in preventing and alleviating various diseases, including diabetes, neuroinflammation, and aging [229].

EGCG, a common polyphenol, could reduce the metabolic disorders induced by a high-fat diet via increasing DNMT1 expression and subsequent CpG loci hypomethylation in the colon, which may be related to EGCG’s activity in reducing the ratio of *Firmicutes* to *Bacteroidetes*. EGCG could inhibit the activity of DNMTs, thereby inhibiting the growth of tumor tissue. Intestinal microorganisms secrete kinds of enzyme systems that are closely related to host energy metabolism, substance metabolism, and other physiological processes, such as hydrolases, oxidoreductases, lytic enzymes, etc. EGCG is metabolized into valeric acid, phenolic acid, and other compounds under the action of these enzymes. The bioavailability of EGCG in vivo is very low and only 0.1–1.6% is absorbed after entering the mouse intestine [230,231]. However, after microbial transformation, the absorption rate could exceed 32.1%, with improved bioavailability [232,233,234]. The intestinal flora of mice with roles in mediating EGCG transformation include *Enterobacter aerogenes*, *Raoultella planticola*, *Adlercreutzia equolofaciens MT4s-5*, *Slackia equolifaciens JCM 16059*, and the human intestinal flora include *Eggerthella SDG-2*, *E. coli*, *Enterococcus*, *Bacteroides*, *Bifidobacterium*, *Lactobacilius*, *lactic acid bacteria*, *acetic acid bacteria*, and *yeast* [235,236,237,238,239,240,241]. The products of EGCG transformation mediated by different microorganisms are not exactly the same. Therefore, these intestinal floras may affect the DNA methylation process of EGCG by affecting its bioavailability and its transformation.

On the other hand, DNA demethylases such as TET proteins and isocitrate dehydrogenase mediate the removal of methyl groups [242,243]. TET methylcytosine dioxygenase oxidizes 5-methylcytosine to 5-hydroxymethylcytosine, forming higher-level oxidation products, which participate in regulating DNA methylation status through the demethylation mechanism [214]. Isocitrate dehydrogenase converts isocitrate into α-ketoglutarate, promoting α-ketoglutarate-dependent dioxygenase, which is involved in DNA demethylation [243]. It was found that SCFAs affected the expression levels of other intermediates in the tricarboxylic acid cycle, such as α-ketoglutarate, fumarate, and succinic acid, by promoting the increase of acetyl-CoA levels in cells, which regulated the enzyme activity of TET methylcytosine dioxygenase [244]. α-Ketoglutarate plays a role as a co-substrate of TET dioxygenase to control the demethylation process, while the TET enzyme is inhibited by fumarate and succinic acid, thereby increasing DNA methylation levels [203]. In IECs of germ-free mice, low levels of the folate cycle induce DNA hypermethylation, which is also associated with a significant loss of TET methylcytosine dioxygenase and reduced DNA methyltransferase activity [245]. Hence, intestinal flora may affect the activities of DNA methylase and DNA demethylase by affecting the synthesis of SCFAs, thereby regulating the DNA methylation status.

### 4.2. Effects of DNA Methylation on Intestinal Flora

In recent years, with in-depth research on intestinal microbiota, it has been found that epigenetic modifications may affect the homeostasis and disordered states of the host by shaping and/or regulating the composition and diversity of the intestinal microbiota, especially under acquired factors such as diet, antibiotic use, and infection. Compared with miRNA, there are fewer studies on how DNA methylation modification affects intestinal flora. The homeostasis of the intestinal environment is mainly maintained by the intestinal microbiota, intestinal mucosal barrier, intestinal immune system, intestinal nervous system, nutrients, etc. These factors rely on the normal expression of host genes. DNA methylation modification affects the function of host genes by regulating their expression, thereby destroying the stability of the intestinal environment.

The intestinal mucosal barrier consists of a mechanical barrier, a chemical barrier, an immune barrier, and a microbial barrier. Each barrier has different structures, molecular regulatory mechanisms, and abilities to influence microbial colonization. At the same time, they are organically combined through their respective signals and jointly maintain intestinal homeostasis [246]. The expression of molecules in the tight junctions between epithelial cells in the mechanical barrier, such as β-catenin and E-cadherin [247,248], is regulated by DNA methylation modification, and DNMT3A maintains the function and regeneration of the intestinal epithelial barrier in the colon [249]. The secretion of gastric acid, bile, hormones, and digestive enzymes in the chemical barrier is also regulated by DNA methylation modification. The development and function of various immune cells (macrophages, dendritic cells, neutrophils, T cells, and B cells) [250,251,252,253] that constitute the intestinal mucosal immune barrier are relevant to DNA methylation. DNA methylation modification directly or indirectly impacts the survival, composition, and normal functions of intestinal flora by acting on these molecules, structures, cells, etc.

## 5. The Correlation and Mutual Regulation between Intestinal Flora, DNA Methylation, and Obesity

The interaction between intestinal flora and DNA methylation may also play an important role in the development of obesity and its complications. Obese individuals tend to exhibit genomic DNA hypomethylation patterns and reduced gut microbiota diversity. After obese mice were fed an HFD, the intestinal flora changed, the abundance of *Firmicutes* and *Actinobacteria* decreased, and the abundance of *Proteobacteria*, *Bacteroidetes*, *Enterobacteriaceae*, and *Klebsiella* increased. Meanwhile, changes in DNA methylation occurred in the promoter region, accompanied by the mRNA expression of adiponectin, resistin, and lipid oxidation-related genes (PPAR-α, PGC-1α, and ATGL) [254]. Furthermore, when there were different proportions of *Firmicutes*/*Bacteroidetes* in the gut microbiota of obese subjects, DNA methylation patterns in blood and adipose tissue were completely different. Moreover, there were significant differences in the methylation levels of 258 genes in blood and adipocytes between the low-ratio group and the high-ratio group, including IGF2BP2 and HDAC7 [255]. Kumar et al. found that infants born to mothers with higher levels of *Firmicutes* in the gut showed DNA methylation changes compared with those born to mothers with higher levels of *Bacteroidetes*, and the differential methylation genes in infants with higher levels of maternal *Firmicutes* were positively correlated with obesity, lipid metabolism abnormalities, inflammation, and cardiovascular diseases [17]. These data indicate that the changes in intestinal flora may affect the expression of genes related to glucose metabolism, energy homeostasis, and lipid metabolism through DNA methylation modification, thereby promoting the occurrence and development of obesity.

Walker et al. found that change in gut microbiota diversity is associated with an increase in BMI, and its process is related to the metabolic disorders caused by DNA methylation [256]. DNA methylation sequencing analysis showed that genes with differentially methylated promoters in pregnant women with abundant *Firmicutes* were related to lipid metabolism, inflammation, and risk of obesity, suggesting that there is a clear association between bacterial dominance and DNA methylation, which might regulate obesity through DNA methylation of obesity-related genes [257]. Polymorphic genes related to obesity, such as PIP5K1A, identified in primary human colonic epithelial cells were differentially expressed after being co-cultured with gut microbiota [258]. Ramos-Molina et al. demonstrated that the composition of gut microbiota in obese patients largely affects DNA methylation status, and the expression levels of genes related to glucose and energy homeostasis (such as HDAC7) may also be regulated by the epigenetic regulation of gut microbiota [255]. Additionally, scholars have found that through effective measures to regulate gut microbiota, the methylation levels of some genes increased, including IL-1β, IL-6, and TNF-α. The above gene expression products are inflammatory factors, which can directly participate in the occurrence and development of obesity.

As gut microbiota metabolites, SCFAs could reduce the expression of DNMT1, DNMT3A and DNMT3B, and methyl-CpG-binding domain protein 2 (MBD2) and inhibit the combination of these enzymes with adiponectin and resistin promoters. The decrease in adiponectin level and the increase in DNA methylation levels in the adiponectin promoter region may be important factors in accelerating the progression of non-alcoholic fatty liver disease (NAFLD). After probiotic treatment, NAFLD rats could reverse this process and simultaneously change *Firmicutes* and *Lactobacillus* abundance [259]. FFAR3 could regulate the cycle of satiety and starvation [260], and the reduction of *Clostridium* and *Plasmodium* could impact the epigenetic regulation of T2DM patients and reduce the methylation of the FFAR gene [260]. SCFAs, as ligands of FFAR2 (GRP43) and FFAR3 (GRP41), play a role in lipid and glucose metabolism [261]. Butyrate could regulate the hypomethylation of the FFAR3 gene and the LINE1 gene, influencing metabolic diseases such as obesity and type 2 diabetes. α-ketoglutarate, an intermediate product of intestinal flora metabolite SCFAs, may regulate the methylation of the Myod1 promoter by affecting the PRDM16 gene, promote beige lipogenesis and thermogenesis, and eventually improve obesity [262,263]. Folic acid supplementation could improve insulin resistance and obesity-related metabolic disorders by affecting the distribution of differentially methylated gene regions in the adipocytes of obese mice and reducing overall methylation levels [264]. Choline is a water-soluble vitamin-like nutrient that can be metabolized into trimethylamine (TMA) by the gut community [265]. TMA is then converted to trimethylamine-N-oxide (TMAO) by flavin monooxygenase (FMO) enzymes, which is associated with an increased risk of obesity and diabetes [171,172,173].

Notably, through a comprehensive analysis of the existing literature, we found that there is an intersection between DNA methylation and gut microbiota in the pathways and mechanisms involved in the occurrence and development of obesity, such as diet, the microbiota–gut–brain axis, adipose tissue development, differentiation and function, lipid metabolism, glucose metabolism, insulin resistance, immune inflammatory response, and Vit D metabolism. Therefore, they may crosstalk and influence each other through these biological pathways and work together on obesity. Dysbiosis of gut microbiota and changes in DNA methylation could contribute to the development of obesity. In turn, metabolic alterations associated with obesity would induce dysbiosis of gut microbiota and changes in DNA methylation.

## 6. Treatment of Obesity Based on Intestinal Flora and DNA Methylation

Targeted anti-obesity interventions, such as diet, exercise, and gastrointestinal surgery, may have an impact on epigenetic processes [266]. It has been proven that metabolic surgery has a long-term effect on multiple DNA methylation sites near more than 400 differentially expressed genes, which are mainly involved in mitochondrial function, glucose and lipid metabolism, and calcium signal transduction [267]. The characteristics of transient, dynamic, and reversible epigenetic changes provide an emerging field for discovering future therapeutic targets in obesity and T2DM [268].

Probiotics, as beneficial bacteria, could change the composition of intestinal flora and regulate the intestinal balance of the host [269,270]. Gut microorganisms are being selected as the next generation of probiotics, and *Lactobacilli* and *Bifidobacteria* have been successfully used to treat obesity [271,272]. Prebiotics are non-digestible food ingredients, such as fructooligosaccharides, galactooligosaccharides, lactulose, and non-digestible carbohydrates, which are called synthetic bacteria when used in combination with probiotics [270]. Studies have found that prebiotics could enhance the secretion of satiety hormones after meals, improve the glucose and fat metabolism of obese and diabetic animals, and improve leptin sensitivity by improving EEC activity [124,125]. Clinical studies have reported that the supplementation of Visbiome^®^ probiotics could reduce fasting blood glucose levels and the ratio of *Firmicutes*/*Bacteroidetes*, thereby improving severe obesity in adolescents [273]. Both in vivo and clinical studies have discovered that prebiotic supplementation with physical exercise could reduce BMI and liver and plasma cholesterol, as well as improving glucose tolerance in obese individuals and mice fed a high-fat diet [274].

FMT could impact the epigenome of host and immune cells, which may provide a new way for the treatment of metabolic syndrome. Compared with autologous FMT, the intestinal flora of the host undergoing allogeneic transplantation is more variable. *Prevotella* is directly associated with the methylation of the AFAP1 gene [275]. In addition, combined treatment with diet and FMT could rapidly increase the abundance of beneficial bacteria such as *Bifidobacterium* in the intestinal flora, which may control blood sugar and reverse dyslipidemia and arteriosclerosis. When subjects with abnormal abdominal obesity were treated with self-FMT through diet regulation, the green MD diet induced significant alterations in the composition of the intestinal flora and better optimized the effects of autologous FMT [276].

Phages could restore dysfunctional colonies, and they show new therapeutic potential in ameliorating ecological imbalance and treating diabetes, obesity, etc. [277]. Fecal virus transplantation (FVT) reduces the risk of invasive infections caused by bacteria by removing bacterial components from donor stool. Studies found that after the phage colony was transferred to the intestine by FVT, the composition of intestinal microbiota could be changed and the symptoms of obesity and T2DM could be alleviated [278]. The identification and isolation of key phages will help develop efficient and personalized phage therapies for complex diseases such as IBD, obesity, and T2DM.

## 7. Conclusions and Prospects

Obesity is a major threat worldwide. Analyzing the relationship between gut microbiota and DNA methylation could provide new clues for the treatment of metabolic diseases. Intestinal flora is increasingly recognized as an environmental factor influenced by DNA methylation. Microbial-derived metabolites, especially SCFAs, as epigenetic substrates, change the structure and status of chromatin, affect the upregulation of metabolism-related gene expression, and reprogram the host transcriptome. However, there are still many limitations in the current research.

In the process of reviewing the literature, we observed that most studies focused on the correlation between intestinal flora and DNA methylation in individuals with obesity, and how gut microbiota participated in the occurrence and development of obesity by affecting DNA methylation. However, there are a few studies on how to directly affect the intestinal flora by regulating overall DNA methylation levels, or the DNA methylation status of specific genes, and then inducing or improving obesity. Therefore, in the future, more in-depth studies are warranted to elucidate the mechanism of DNA methylation in modulating gut microbiota and how this mechanism affects obesity.

Furthermore, although existing studies indicated that there were correlations between obesity and DNA methylation, as well as between obesity and gut microbiota, establishing causality in these relationships poses a significant challenge. The reciprocal relationship between alterations in gut microbiota and DNA methylation may contribute to the pathogenesis of obesity. Consequently, further investigations should utilize more robust experimental designs to elucidate and validate the causal mechanisms underlying these associations, thereby enhancing the efficacy of obesity management and prevention strategies.

In the context of obesity, it is widely recognized that both DNA methylation and gut microbiota play crucial roles in its etiology and progression. A deeper investigation into the intricate relationship and underlying mechanisms linking obesity with gut microbiota and DNA methylation holds promise for advancing therapeutic strategies. Furthermore, the interaction between DNA methylation and gut microbiota should not be overlooked in the pathogenesis of obesity, and exploring the specific molecules and signaling pathways involved in this interaction is crucial for the development of innovative therapeutic and preventive approaches for obesity.

## Data Availability

All reports supporting the discussion are available in this paper.
